# Time-course of hemispheric preference for processing contralateral relevant shapes: P1pc, N1pc, N2pc, N3pc

**DOI:** 10.2478/v10053-008-0098-9

**Published:** 2012-02-03

**Authors:** Rolf Verleger, Blandyna Żurawska vel Grajewska, Piotr Jaśkowski

**Affiliations:** 1 Department of Neurology, University of Lübeck, Germany; 2 Faculty of Psychology, University of Finance and Management, Warsaw, Poland

**Keywords:** N2pc, attention, event-related potentials, P1pc, N1pc, N3pc, SPCN

## Abstract

A most sensitive and specific electrophysiological indicator of selective processing of visual stimuli is the N2pc component. N2pc is a negative EEG potential peaking 250 ms after stimulus onset, recorded from posterior sites contralateral to relevant stimuli. Additional deflections preceding or following N2pc have been obtained in previous studies, possibly produced by specific stimulus features or specific prime-target sequences. To clarify the entire time-course of the contralateral- ipsilateral (C-I) difference recorded from the scalp above visual cortex in response to left-right pairs of targets and distracters, C-I differences were here compared between two types of stimuli and between stimuli that were or were not preceded by masked neutral primes. The C-I difference waveform consisted of several peaks, termed here *P1pc* (60-100 ms after target onset), *N1pc* (120-160 ms), *N2pc* (220-280 ms), and *N3pc* (360-400 ms). Being markedly enhanced when stimuli were preceded by the neutral primes, P1pc may indicate a response to stimulus change. Also, when stimuli were primed, N2pc reached its peak earlier, thereby tending to merge with N1pc. N3pc seemed to increase when target discrimination was difficult. N1pc, N2pc, and N3pc appear as three periods of one process. N3pc probably corresponds to L400 or SPCN as described in other studies. These observations suggest that the neurophysiological basis of stimulus-driven focusing of attention on target stimuli is a process that lasts for hundreds of milliseconds, with the relevant hemisphere being activated in an oscillating manner as long as required by the task.

## Introduction

When two stimuli are simultaneously presented left and right from fixation and the electroencephalogram (EEG) is recorded from the observer’s scalp, then the more relevant stimulus will evoke a negative EEG potential at the scalp above the contralateral visual cortex, peaking at about 250 ms after stimulus onset (e.g., Eimer, 1996; Hickey, Di Lollo, & McDonald, 2009; Hopf et al., 2006; Wascher & Wauschkuhn, 1996). This potential was termed *N2pc* by Luck, Fan, and Hillyard (1993), that is, a negative peak in the time range of the N2 peak, posterior contralateral. N2pc presumably indicates selective processing of the relevant feature (Eimer, 1996; Eimer & Kiss, 2008; Verleger & Jaśkowski, 2007; Wauschkuhn et al., 1998). N2pc may be conveniently measured in the difference waveform contralateral minus ipsilateral (CI) to the relevant stimulus because everything common to the relevant and irrelevant stimuli will then be subtracted out, leaving what is specific to relevance (Oostenveld, Stegeman, Praamstra, & van Oosterom, 2003; Wascher & Wauschkuhn, 1996).

Being larger contralateral to the relevant stimulus, N2pc faithfully reflects the side on which the observer perceived something relevant. This feature made N2pc a useful tool for disentangling brain responses to stimuli presented in close succession, as for example target stimuli immediately followed by some mask, target stimuli immediately preceded by some priming stimuli, or target stimuli embedded within series of distractors, because the side specificity may provide a unique trace of either of these stimuli (Jaśkowski, Skalska, & Verleger, 2003; Jaśkowski, van der Lubbe, Schlotterbeck, & Verleger, 2002; Verleger & Jaśkowski, 2007; Verleger, migasiewicz, & Möller, 2011; Woodman & Luck, 1999).

**Figure 1.**
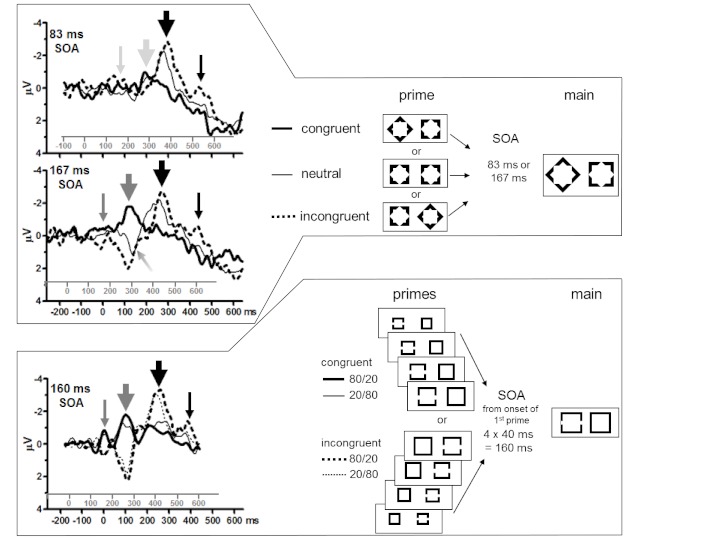
Contralateral-ipsilateral grand averages |P7-P8| and stimuli from Jaśkowski, van der Lubbe, and Schlotterbeck (2002) (top) and Jaśkowski, Skalska, and Verleger (2003) (bottom). The sequence of stimuli in a trial is depicted on the right side of the figure, exemplified for targets presented left of fixation (diamonds and squares with gaps). Primes could be congruent (target shape on the same side as in the main stimulus), or incongruent (target shape on the other side), or neutral (in the diamond experiment only). In the gap-square experiment (lower panel), all four primes had the gap-square on the same side in a given trial and, across blocks, congruent and incongruent trials had frequencies either of 80/20% or of 20/ 80%. In the grand averages (of 12 and 11 participants for the two studies, respectively), the x-axes denote milliseconds from (1^st^) prime onset (gray) and from target onset (black). y-axes are in microvolts, with negative polarity contralateral to position of target plotted upwards. In the diamond experiment, bold solid lines are from congruent trials, thin lines from neutral, and dotted lines from incongruent trials. In the gap-square experiment, solid lines are from congruent trials, dotted lines from incongruent trials, and bold lines are from the 80/20 block, thin lines from the 20/80 block. Thin gray vertical arrows point to N160pc evoked by target-like shapes in the prime stimuli. Big gray arrows highlight N2pc evoked by target-like shapes in the prime stimuli. (Both gray arrows are depicted in lighter gray in the upper panel, reflecting the absence of these deflections.) Big black arrows highlight N2pc evoked by targets in the main stimuli. Thin black arrows point to N400pc evoked by targets in the main stimuli. The sloping arrow (middle panel) points to a peak that may be interpreted either as P300pc evoked by neutral primes or as P130pc evoked by targets preceded by neutral primes. SOSOA = stimulus onset asynchrony between onset of (first) prime and main stimulus.

However, this specificity of N2pc to side of the relevant stimuli does not resolve all difficulties of interpreting overlap of components in the C-I difference. Thus, the present study was prompted by questions raised from considering and comparing the results of two studies of ours (Jaśkowski et al., 2002, 2003). In both studies, metacontrast-masking was used, with stimuli presented as primes whose outer contours fit the inner contours of the ensuing visible main stimuli. Jaśkowski et al. (2002) used diamonds and squares with octagonal inner contours (cf. Klotz & Wolff, 1995) while Jaśkowski et al. (2003) used simple squares. These stimuli and the grand means of the C-I difference waveforms are depicted in Figure 1. In both studies, participants had to ignore the primes and make speeded left vs. right choice responses according to the side of the target in the main stimuli. (The visible stimulus pair, which included the target as one of its two stimuli, will be called *main stimuli* throughout.) Both studies focused on N2pc evoked by the target in the main stimuli (black big arrows in Figure 1) and varying as a function of the primes, and on N2pc evoked by the target-like shape in the prime stimuli (gray big arrows in Figure 1). One obvious difference in the latter, prime-evoked N2pc between studies was that in Jaśkowski et al. (2002), N2pc was evoked when primes were weakly identifiable (middle panel of Figure 1) but not reliably when primes were unidentifiable (denoted by lighter gray of the big arrow in top panel of Figure 1), whereas a distinct N2pc was evoked by unidentifiable primes in Jaśkowski et al. (2003; see gray big arrow in bottom panel of Figure 1). Second, there was a conspicuous prime-evoked component at 160 ms already (cf. thin gray vertical arrows in Figure 1), both in Jaśkowski et al. (2003) and, less distinctly, in the 167 ms SOA (stimulus-onset asynchrony) condition of Jaśkowski et al. (2002) and not in the 83 ms SOA condition (denoted by lighter gray of the thin gray vertical arrow in top panel of Figure 1). The question may be asked whether this early peak is also evoked by the following visible target stimuli, remaining unnoticed because of overlapping potentials evoked by the prime. Third, targets did not only evoke the N2pc but also a consistent though weak later negative peak at about 400 ms after main-stimulus onset (thin black vertical arrows in Figure 1).^1^ This raises the question of how reliable this peak was. Fourth, when primes did not contain the target shape (i.e., were neutral) and were identifiable, a positivity was evoked in the C-I waveform at about 130 ms after target onset and about 300 ms after prime onset (sloping arrow in middle panel of Figure 1). It was unclear what this deflection reflected.

These differences between the two studies might be due to differences between stimuli (diamond and square with complex inner contour in one experiment vs. simple square with and without gaps in the other), due to differences between the priming sequences (one prime, either at 83 ms or at 167 ms, in one experiment vs. a sequence of four primes, at 160 ms, 120 ms, 80 ms, 40 ms, in the other experiment) or due to some other characteristics. The purpose of the present experiment was to clarify these issues by focusing on the difference between stimuli and between primed and unprimed sequences. With regard to stimuli, we were interested to see if and how the C-I waveforms would differ between selection for diamonds versus squares and selection for gap-squares versus intact squares. In particular, squares with gaps might evoke an earlier contralateral negativity, at 160 ms, like in Jaśkowski et al. (2003), whereas diamonds might not. Second, with regard to primes, we were interested in how the mere presence of primes would alter these effects. Therefore, primes were always neutral in the present study, so as not to prime specific responses or activate attentional focusing on one side, but rather to obtain their alerting and spatial orienting effects simultaneously on both lateral positions (Fan, McCandliss, Sommer, Raz, & Posner, 2002; van der Lubbe, Keuss, & Stoffels, 1996). In comparing the two types of stimuli and their prime sequences, the difficulty arose that the timing of the prime sequences differed between the two studies: Primes were well masked with 83 ms prime-main SOA in Jaśkowski et al. (2002) and with 40 ms SOA for each of the intervals of the five prime-prime-prime-prime-main stimuli in Jaśkowski et al. (2003; cf. the stimulus sequences in the right half of Figure 1). In order to compare these stimulus sequences under conditions that were as equal as possible to each other on the one hand and to the original stimuli on the other hand, we decided to use the same SOA of 80 ms for both types of stimuli between onsets of prime and of main stimulus. This meant reducing the number of primes from four to two with the gap-square sequence while maintaining the original 40 ms SOA. We did not use only one prime with these stimuli, with an 80 ms SOA before the main stimulus, because we assumed that this longer SOA would alter prime visibility compared to Jaśkowski et al.’s (2003) original study.

Earlier peaks than N2pc, as found here at 160 ms after primes, have been occasionally reported in the C-I waveform. For example, when presenting relevant stimuli and symmetrically positioned filler stimuli, both Valle-Inclán (1996) and Wascher, Schatz, Kuder, and Verleger (2001) obtained the usual N2pc with a maximum at 250 ms, which changed to an asymmetry of the earlier N1 component in their control experiments where the filler stimuli were omitted. This issue was more systematically reconsidered by Wascher and Beste (2010). These authors obtained both N2pc and earlier N1pc components. The N1pc was assumed to reflect initial orienting of attention, and the N2pc to reflect a reallocation process following initial orienting.

Contralateral negativities later than N2pc in the C-I waveform have been more often described, sometimes as a peak (e.g., L-400 of Wauschkuhn et al., 1998), sometimes as a tonic surplus of contralateral negativity (Vogel & Machizawa, 2004), and have been called *sustained posterior contralateral negativity* (SPCN; cf. Dell’Acqua, Sessa, Jolicoeur, & Robitaille, 2006; Jolicoeur, Sessa, Dell’Acqua, & Robitaille, 2006) or *contralateral delayed activity* (CDA; Gao, Yin, Xu, Shui, & Shen, 2011; Mazza & Caramazza, 2011). The tonic component has been shown to reflect load and individual capacities of visual working memory (Vogel & Machizawa, 2004; Vogel, McCollough, & Machizawa, 2005) but has also been obtained without any obvious relation to working memory, for instance, when the two searched-for targets were in the same hemi-field (Mazza & Caramazza, 2011; Woodman & Luck, 1999) or when the salient stimulus was indeed the relevant one (Seiss, Kiss, & Eimer, 2009; Wauschkuhn et al., 1998), probably just reflecting ongoing re-levant stimulation from the same hemi-field.

Here, we wanted to study whether these components earlier and later than N2pc may also be obtained in simple choice-response tasks and how they depended on the physical nature of stimuli and on the presence of preceding neutral primes.

## Methods

### Participants

Ten students (aged 19-22 years; one man, nine women) of the University of Bydgoszcz, participated in return for course credit. They were seated in a comfortable chair, with 0.75 m eye-distance to the screen. The keyboard for responding was placed on a table in their front.

### Stimuli and procedure

**Figure 2.**
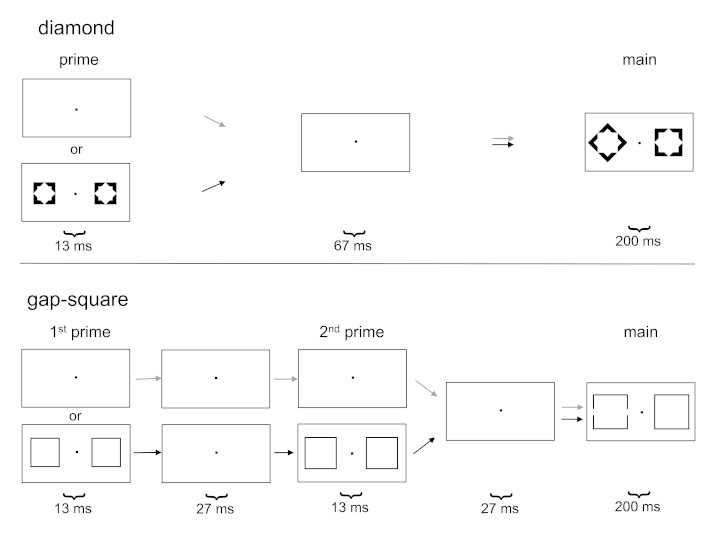
Stimuli used in the present experiment. Targets were in different blocks either diamonds or squares with gaps, in random order either on the left side of the main stimulus (as in the figure) or on the right side. In a random half of the trials, stimuli were preceded by primes, which always consisted of pairs of non-target squares.

Black stimuli, as depicted in Figure 2, were presented on a 21’’ white screen driven by a graphics card working with 75 Hz. A fixation dot (0.3° diameter) was displayed throughout. In each trial, a pair of target and distracter stimuli was presented, one left and the other one right of fixation for 200 ms (“main stimulus”). Targets were left or right, in random order across trials. According to target side, participants had to press the left or the right key (left Control key or Enter key of the number block) on a keyboard. The main stimuli were preceded by primes in a random half of trials. Primes always consisted of a pair of stimuli slightly smaller than the main stimuli, and were presented for 13 ms, to be masked by metacontrast, like in Jaśkowski et al. (2002, 2003). By always having the shape of distracters, the two primes were neutral with respect to the response required to the target. Centers of all stimuli were 2.3 cm (1.8°) away from fixation. Each new trial started 840 ms after participant’s key-press.

In one block, the distracter was a square, 2.2 cm wide and high (1.7°), and the target was a diamond (i.e., a square rotated by 45°). Outlines of the shapes were 0.3 cm wide, and inner contours of these outlines were octagonal, matching both a diamond and a square, as used in metacontrast studies since Klotz and Wolff (1995). Prime sti-muli consisted of a pair of smaller squares, 1.6 cm wide and high, likewise with octagonal inner contours, and were presented 80 ms before the main pair. The primes were as in Jaśkowski et al. (2002).

In the other block, the target was a square with gaps, and the distracter was an intact square. Squares were 2.6 cm (2.0°) wide and high, drawn with 0.1 cm wide lines. The gaps were 0.3 cm (0.25°) wide, in the middle of the vertical outlines of the target square. Prime stimuli consisted of two successive pairs of intact squares. The first pair was presented 80 ms before the main pair and was 2.2 cm wide and high, the second pair was presented 40 ms later and was 2.4 cm wide and high. This multiple-prime sequence of stimuli was like the stimulus sequence used by Jaśkowski et al. (2003) except for the fact that four pairs of primes had been used in that study.

There were 400 trials in either block. Order of blocks alternated between participants.

### EEG recording and processing

EEG was recorded from 10 scalp sites (Fz, Cz, Pz, Oz, C3, C4, P7, P8, PO7, PO8) with Ag/AgCl electrodes positioned in an elastic cap (FMS, Munich). An electrode at the nose served as common reference, a forehead electrode as ground. Electrooculogram (EOG) was recorded for controlling intrusion of ocular potentials into the EEG from above and below the right eye and from the outer canthi of both eyes. EEG and EOG were amplified from 0.03 Hz to 100 Hz and stored at 250 Hz per channel by a *BrainAmp* amplifier. Off-line, data were low-pass filtered at 20 Hz, segmented from 200 ms before onset of the main stimulus to 900 ms afterwards, and edited for artifacts (rejecting trials with zero lines, followed by correcting ocular artifacts, followed by rejecting trials with voltage differences within the trial of more than 200 µV or voltage steps from one data-point to the next of more than 20 µV). The first 100 ms (200 ms 100 ms before onset of the main stimulus) were used as baseline. To obtain averaged contralateral-ipsilateral difference potentials separately for each condition and participant, data were averaged across artifact-free trials in which participants correctly responded, separately for trials with left- and right-side targets. Next, the contra-ipsilateral difference was formed in either average (e.g., for PO7 and PO8: PO8 PO7 for the left-side average and PO7 PO8 for the right-side average) and these two differences were averaged (termed |PO7 PO8|). Grand means over participants were calculated for illustrating the results.

### Data analysis

Response times were measured by using the response-triggered markers set by the control program onto the EEG recording and referring these values to target onset. Mean response times were calculated as averages across correct responses. Percentages of correct responses were determined as ratios of correct responses relative to all trials. Repeated-measures analyses of variance (ANOVAs) were computed with two 2-level factors: Stimulus (gap-square vs. diamond) and Prime (absent, present).

For an analysis of the |PO7-PO8| waveforms averaged for each participant and condition, mean amplitudes were formed over successive 20 ms windows from target onset to 600 ms afterwards. Each window’s activity was analyzed with an ANOVA with the same design as for behavior (2 stimuli × 2 prime conditions). The analysis first focused on whether activity significantly deviated from baseline across all four stimulus and prime conditions. This comprehensive analysis was extended by *t*-tests for deviation from zero, separately for each of the four conditions. Then, effects of the Stimulus and Prime factors were analyzed. In addition to the 20 ms windows, peaks of the deflections were determined, as described in the Results, and their latencies were submitted to the Stimulus × Prime ANOVA.

Finally, in addition to the |PO7-PO8| waveforms, nose-referenced waveforms of the visual evoked potential recorded at PO8 were analyzed and compared to the |PO7-PO8| waveforms.

Degrees of freedom in ANOVAs were 1/9 throughout.

## Results

### Behavior

Mean response time was 386 ms and mean error rate was 5.7%. Primed responses were 15 ms faster than unprimed responses, *F* = 12.3, *p* = .007, without difference between the two stimuli (Prime × Stimulus: *F* = 1.5, *ns*; main effect of Stimulus: *F* = 0.6, ns). The faster responses after primes were more error-prone, with 3.4% more errors committed in primed than unprimed responses (*F* = 10.8, *p* = .009). Thus, the non-informative primes obviously lowered the criterion for responding. Further, diamonds led to 3.2% more errors than gap-squares (*F* = 10.2, *p* = .01). Apparently, diamonds and squares with their complex and equal inner contours were harder to discriminate from each other than were thin gap-squares from squares. The interaction was not significant (*F* = 0.4).

### EEG potentials

**Figure 3.**
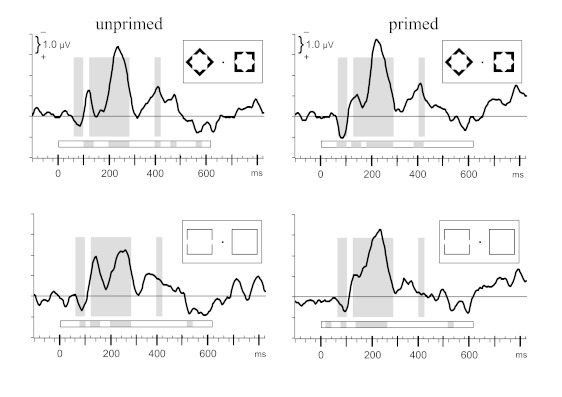
Grand means (*N* = 10 participants) of contra-ipsilateral waveforms recorded from |PO7 − PO8|. Upper panels are from blocks with diamonds as targets, lower panels from blocks with gap-squares. Left panels are from unprimed trials, right panels from trials where the main stimuli were preceded by primes (prime onset at -80 ms). x-axes denote milliseconds from onset of main stimuli (0 ms), with small ticks every 20 ms. y-axes are in microvolts, with negative polarity contralateral to position of target shape plotted upwards and ticks in 1 μV intervals. Gray shadings denote time windows where deviation from baseline was significant as a main effect across all four panels. Grey shades in the horizontal bars denote time windows where deviation from baseline was significant for the waveform depicted in the particular panel.

Grand means of the C-I difference waveforms at |PO7 PO8| are displayed in Figure 3. Windows in which the ANOVA’s constant term differed from zero are illustrated in Figure 3 by gray background shading underlying the waveforms (equal for all four waveforms), and windows where *t*-tests displayed deviations from baseline for a given condition are marked by shading in the horizontal bars displayed beneath each waveform. As is evident, there was an early contralateral positivity from 60 ms to 100 ms (activity in the two windows 60-80 ms and 80-100 ms: *F* ≥ 11.1, *p* < .009). Then, three periods of contralateral negativity occurred: the first one at 120160 ms (*F* ≥ 12.4, *p* < .007), which was followed after a plateau phase (160-200 ms: *F* ≥ 9.2, *p* < .01) by a second, longest period at 200-280 ms (200-260 ms: *F* ≥ 49.2, *p* < 260-280 ms: *F* = 18.9, *p* = .002), and a third one at about 360400 ms (380400 ms: *F* = 7.4, *p* = .02), with *t*-tests being significant in this third period for diamonds only. In shorthand notation, the early contralateral positivity will be called *P1pc*, and the following three periods of negativity will be called *N1pc*, *N2pc*, and *N3pc*.

**Figure 4.**
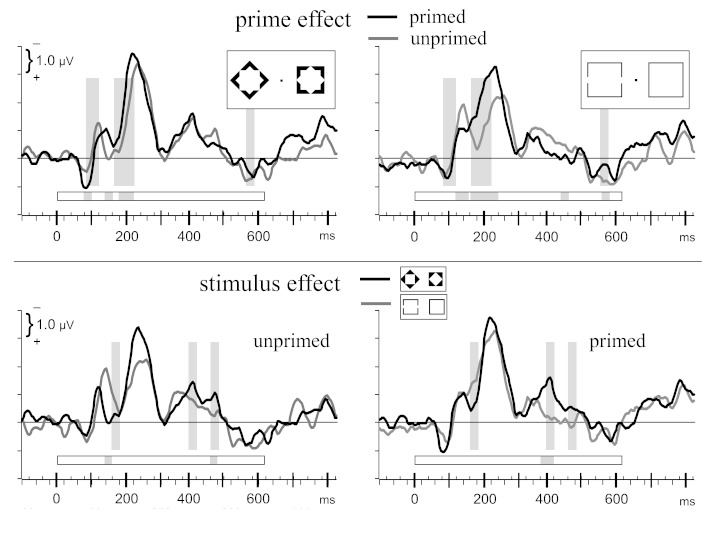
Rearrangement of the waveforms depicted in Figure 3, in two ways. In the upper panels, primed and unprimed waveforms are overlaid (left and right panels of Figure 3). Gray shadings denote time windows where the main effect of prime was significant across stimuli. Gray shades in the horizontal bars denote time windows where primed and unprimed waveforms differed in the particular panel. In the lower panels, waveforms are overlaid from diamonds and gap-squares (upper and lower panels of Figure 3). Gray shadings denote time windows where the main effect of stimulus was significant across primed and unprimed waveforms. Gray shades in the horizontal bars denote time windows where waveforms differed between diamonds and gap-squares in the particular panel. x-axes denote milliseconds from onset of main stimuli (0 ms), with small ticks every 20 ms. y-axes are in microvolts, with negative pola-rity contralateral to position of target shape plotted upwards and ticks in 1 μV intervals.

The following effects of priming were noted in the ANOVAs (see top panels of Figure 4, where the primed and unprimed waveforms from Figure 3 have been overlaid). P1pc was larger with primed than with unprimed diamonds (80-100 ms: main effect of Prime: *F* = 5.7, *p* = .04; Prime × Stimulus: *F* = 5.0, *p* = .052) delaying the rise of N1pc (100-120 ms: main effect of Prime: *F* = 7.5, *p* = .02). The following Prime × Stimulus interactions (120-140 ms: *F* = 5.9, *p* = .04; 140-160 ms: *F* = 28.7, *p* < .001) on the one hand indicated this delayed rise of N1pc with primed diamonds (thereby producing more negativity for primed than for unprimed diamonds in this period) and, on the other hand, indicated that N1pc was larger for unprimed than for primed gap-squares. A second effect of priming, on N2pc, started at 160 ms and lasted until 240 ms: N2pc was larger with primed stimuli, due to an earlier rising or larger amplitudes (main effect of Prime: 160-200 ms: *F* ≥ 24.6, *p* < ≤ .001; 200-220 ms: *F* = 13.2, *p* = .005; for gap-squares also at 220-240 ms: Prime × Stimulus: *F* = 5.9, *p* = .04). Finally, of less interest, there was a main effect of Prime at 540-560 ms (*F* = 14.0, *p* = .005). Thus, there were two major effects of priming: P1pc was larger, affecting the following N1pc, and N2pc rose earlier and/or was larger.

There were only a few though possibly interesting differences between stimuli, as illustrated in the bottom panels of Figure 4. N1pc was larger with gap-squares than with diamonds, with unprimed stimuli at 140-160 ms already (resolving the above-noted Prime × Stimulus interaction to an effect of stimulus in unprimed trials, *F* = 15.5, *p* = .003) and generally at 160-180 ms (main effect of Stimulus: *F* = 6.5, *p* = .03). In contrast, N3pc was larger with diamonds than with gap-squares (main effect of Stimulus: *F* = 12.7, *p* = .006 at 380-400 ms; *F* = 5.5, *p* = .04 at 440-460 ms), and at 380-400 ms more so when primed (Stimulus × Prime: *F* = 13.6, *p* = .005). No effects of Stimulus were significant during the large N2pc (different from what inspection of Figure 4 might suggest).^2^

To obtain measures that are more sensitive to differences in timing, peak latencies of the described components were determined. P1pc was defined as the most positive peak 50-125 ms post-stimulus. It was earlier with diamonds (83 ms) than with gap-squares (94 ms; Stimulus: *F* = 9.7, *p* = .01). The first negative peak, N1pc, had its mean latency at 135 ms (range 105-190 ms) without significant differences between prime conditions and stimuli. N2pc varied between 185 ms and 280 ms and peaked earlier with primed than with unprimed stimuli (226 ms vs. 240 ms; Prime: *F* = 6.7, *p* = .03). N3pc varied between 290 ms and 470 ms and peaked earlier with gap-squares than with diamonds (352 ms vs. 375 ms; Stimulus: *F* = 7.3, *p* = .02).

The only effect on amplitudes of these peak measures was on N1pc amplitude which was largest with unprimed gap-squares (Prime × Stimulus: *F* = 9.4, *p* = .01). The prime effect on P1pc amplitude with diamonds, which was significant with the mean-amplitude measure, only tended to significance with peak amplitude (*p* = .06 for main effect of Prime; *p* = .10 for the interaction of Prime × Stimulus). No effect was significant on N2pc and N3pc peak amplitudes.

**Figure 5.**
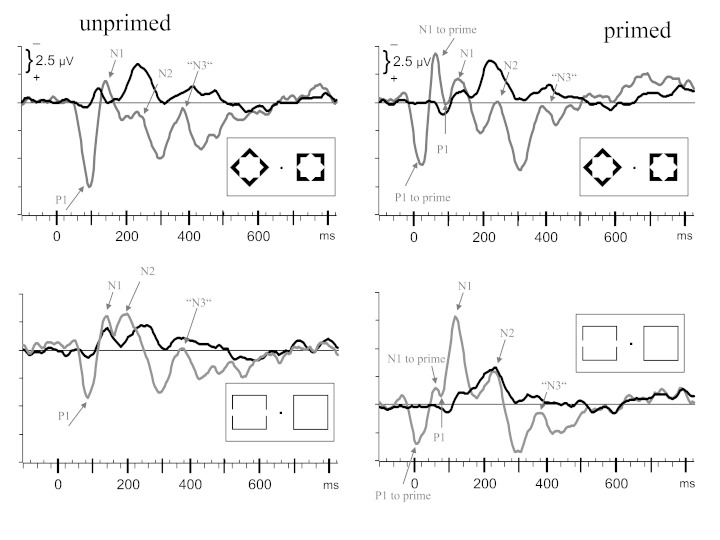
Grand means recorded from PO8 (gray), overlaid on grand means of contra-ipsilateral waveshapes recorded from |PO7 − PO8|, separately for each condition. Conditions are arranged like in Figure 3. Tick marks on y-axes denote 2.5 μV intervals (different from Figure 3).

To provide some relationship between the C-I differences and the usual ERP waveforms, Figure 5 displays recordings from the PO8 site against the nose reference, together with the C-I differences. The nose-referenced recordings show the usual visually evoked potentials, starting with the P1-N1 complex. (Note that in primed trials, there is first a P1-N1 complex to the prime, and the relevant consecutive P1-N1 complex to the main stimulus is grossly altered, much more than the C-I waveforms.) Comparing C-I differences to the nose-referenced recordings shows that all components of the C-I difference-waveform were roughly time-locked to some conventional component, but when looked at in detail there seemed to be relevant differences: P1pc roughly coincided with the P1 component evoked by the main stimuli. But the match was better with squares, whereas with diamonds, P1pc occurred with the first, positive-going phase of P1, therefore peaked earlier than P1. N1pc coincided with the N1 component evoked by the main stimuli, except for unprimed diamonds, where N1pc peaked earlier than N1. N2pc had a good match with some minor N2 component seen in the PO8 waveforms, except for unprimed gap-squares where N2 occurred earlier. N3pc was consistently aligned with some negative N3 peak.

## Discussion

We delineated the entire C-I difference waveform evoked by pairs of target and distracter stimuli presented left and right from fixation. This C-I waveform indicates the extent of preference for the side of the relevant stimulus at any given moment. In addition to the well-known N2pc component, three other deflections could be discerned, which we called *P1pc*, *N1pc*, and *N3pc*.

Of primary interest were differences between stimuli. Attempting to resolve conflicts between previous results (Jaśkowski et al., 2002, 2003), we used pairs of diamonds and squares with octagonal inner contours in one block, and of gap-squares and simple squares in the other block. Indeed, there was a difference between these pairs of well visible main stimuli (lower panels of Figure 4): Gap-square targets evoked larger C-I differences than diamond targets in the first negative deflection, N1pc, and smaller C-I differences in the final deflection, N3pc. Since gap-squares were somewhat easier to identify than diamonds, as indicated by percentages of erroneous key-presses, this result suggests that stimuli that are easier to identify evoke more early and less late contralateral negativity than stimuli that are hard to identify.

The second independent variable was the presence or absence of neutral primes. The major effects of primes were to enhance P1pc and to accelerate the rise of N2pc (upper panels of Figure 4).

Few studies have so far noticed and described the P1pc, N1pc, and N3pc components. The components will be discussed in the temporal order of their occurrence.

### P1pc

P1pc was well visible in the data of Jaśkowski et al. (2002; see Figure 1, sloping arrow) but at that time we were uncertain how to interpret this effect, not being aware of previous occurrences of P1pc in published literature. Yet actually a similar effect on P1 was already obtained in the ground-breaking study by Luck and Hillyard (1994) ascribed by those authors to neuronal dishabituation (because the target stimulus was less frequent than the distracters) rather than to relevance. This view has more recently been resumed and elaborated by Kimura and colleagues (e.g., Kimura, Katayama, & Murohashi, 2006, 2008). In line with this view, data displayed in Wauschkuhn et al. (1998, their Figure 4) show a dissociation between P1pc and N2pc: In each trial, a gray circle was presented opposite to a colored circle, which was either red or blue. In different blocks, either the gray circles or the colored circles were defined as targets. N2pc was always negative contralateral to the *relevant circle*. In contrast, P1pc was always (and significantly, as shown by present reanalysis of those data) positive contralateral to the *colored* circle, thus was affected either simply by color salience or by the change of color between trials (red or blue, whereas gray was presented in each trial). This is in line with Luck and Hillyard’s (1994) dishabituation assumption. The enhancing effect of neutral primes on P1pc in the present study is in line with those findings, reflecting the difference of the target shape from the preceding prime squares. Based on this, it makes sense that P1pc after neutral primes in Jaśkowski et al. (2002; see Figure 1) was specific to neutral primes, because when primes were congruent there was no change at all, and when primes were incongruent there was change on both sides, yielding a zero effect in the C-I difference.

### N1pc

N1pc, as noted in the Introduction, is, trivially, obtained when stimulation is unilateral (Valle-Inclán, 1996; Wascher et al., 2001; Wascher & Beste, 2010) but there are several papers where N1pc was reliably obtained with bilateral stimulation as well. It appears instructive to compare the results of Praamstra and Plat (2001), Praamstra and Oostenveld (2003), van der Lubbe and Verleger (2002), and Experi-ment 3 of Wascher et al. (2001), all of whom obtained a well-delimited N1pc, to the results of Wascher and Wauschkuhn (1996); van der Lubbe, Jaśkowski, Wauschkuhn, and Verleger (2001); and Experi-ment 1 of Wascher et al. (2001) all of whom did not. At first sight, this diffe-rence cannot be due to stimulus material and task, because all those studies used the same material (the letters A and B, presented left or right, opposite from fixation and a “filler” consisting of three horizontal lines) and the same task (left-right choice-responses to A vs. B). Also at second sight, we did not find a variable that could account for these differences: The two types of results could be distinguished neither by speed of responses, nor by color, size, or eccentricity of stimuli; nor by features of the fixation cross or of screen background; nor by length of intertrial interval, nor by participants’ age.

The present data suggest that N1pc is enhanced by particular sti-muli (gap-squares in the present case) and may be better distinguished as a component of its own when there is no preceding prime, because N1pc formed a well-defined peak with unprimed stimuli only, possibly because priming accelerated the N2pc, merging it with N1pc. One might suspect that the biphasic form of N1pc and N2pc in the present grand averages is an artifact of averaging across participants: Possibly some have an N1pc, others an N2pc. Yet, inspection of individual data unambiguously showed this biphasic structure (and even triphasic, including N3pc) as a prevailing pattern in many participants. One might further suspect that this pattern in individuals is an artifact of averaging across trials: Possibly some trials have an N1pc, others an N2pc. To tackle this suspicion, one may dichotomize data according to response times and expect that fast-response trials would be characterized by N1pc, slow-response trials by N2pc. When we did this analysis in the present data (results not reported), this expectation was not borne out, nor was it the case when we reanalyzed some older data (from Wascher et al., 2001).

Praamstra and Oostenveld (2003) assumed, as did Jaśkowski et al. (2003), that N1pc was due to asymmetries of their stimulus material, as a low-level exogenous effect. In line with this, participants’ strategy induced by frequency variation of congruent and incongruent primes affected N2pc but not N1pc in the study by Jaśkowski et al. (2003; the effect is displayed above, as the difference between bold and thin lines in the lower part of Figure 1). On the other hand, while certainly being a necessary condition for N1pc to emerge, physical differences do not explain why N1pc has the same polarity as N2pc: If it was due to physical difference only, the negative deflection might be evoked on the other side as well. Therefore relevance appears to be a decisive factor. Thus, at present, the best guess is that N1pc may be affected both by exogenous asymmetries and by factors that also affect N2pc.

### N3pc

N3pc did not show up as distinctly in the present data as N1pc and N2pc. However, it reliably differed from zero, differed between stimuli (being larger with diamonds than with gap-squares). and furthermore could be well distinguished in many participants’ individual averages, forming a triphasic pattern with N1pc and N2pc. It seems that latency variation between participants made this component appear flatter in the grand mean. N3pc can also be unambiguously seen in Experiment 1 of Wascher et al. (2001, their Figures 2 and 3, “collapsed data”). In one earlier paper of ours, Wauschkuhn et al. (1998), N3pc was analyzed (termed *L400*, by its peak latency) and found to be more selective than N2pc (*L250*): Whereas N2pc was always evoked by relevant stimuli, N3pc was only evoked if these stimuli were also targets of the saccade defined by the relevant stimulus (similarly: Mazza, Turatto, Umiltŕ, & Eimer, 2007; Seiss et al., 2009). N3pc might have been overlooked in previous data, often being pulled below baseline by its riding on the posterior contralateral positivity that is evoked by manual key-presses, for example, in Wascher and Wauschkuhn (1996, their Figure 2).

### N1pc, N2pc, N3pc

N2pc probably reflects attentional capture by relevant stimuli (Eimer & Kiss, 2008; Lien, Ruthruff, Goodin, & Remington, 2008; Seiss et al., 2009). The present data suggest that this capture by and selection of relevant stimuli might proceed in several cycles. The differences that were obtained between gap-square and diamond stimuli may mean that stimuli that are easier to identify evoke more N1pc and less N3pc than stimuli that are hard to identify. Thus, a single pass may be sufficient if stimulation is unilateral, producing N1pc, but multiple passes may be needed if the decision has to be made on the basis of features of bilateral stimuli, producing the periodic structure of N1pc, N2pc, and N3pc.

## Footnotes

1 The assumption that this component reflected target-elicited activity was based on the observation that its peak was constant with respect to target onset (black x-axes) and shifted with respect to prime onset (gray x-axes) by the difference of about 80 ms between prime-target interval conditions depicted in the upper versus middle panel.

2 One referee of this paper argued that differences between stimuli might as well reflect differences between the two different prime sequences: One prime with the diamond-square stimuli, two primes with the gap-squares. Two points can be made about this argument: First, it appears reasonable to assume that these differences in prime sequence would not play any role in no-prime trials. But the early N1pc diffe-rence between stimuli was more marked in unprimed than in primed trials. Second, it appears plausible (though admittedly not compelling) that such effects of the physical prime structure should produce early effects if any. Thus, it appears not probable that the late N3pc effects were produced by those differences.

## Author Note

Piotr Jaśkowski, our friend, colleague, and teacher, passed away on January 6, 2011. Blessed be his memory.

In writing this paper, Rolf Verleger was supported by grant no. Ve110/15-1 from Deutsche Forschungsgemeinschaft, awarded as part of the package proposal *Neuro-Cognitive Mechanisms of Conscious and Unconscious Visual Perception*.
